# Whole blood chromium concentration is very rarely elevated independently of whole blood cobalt

**DOI:** 10.1038/s41598-021-91512-9

**Published:** 2021-06-11

**Authors:** Olli Lainiala, Mari Karsikas, Aleksi Reito, Antti Eskelinen

**Affiliations:** 1 Coxa Hospital for Joint Replacement and Faculty of Medicine and Health Technologies, Tampere University, Niveltie 4, 33520 Tampere, Finland; 2 Imaging Centre, Department of Radiology, Tampere University Hospital, Kuntokatu 2, 33520 Tampere, Finland; 3 Center for Life Course Health Research, University of Oulu, P.O.Box 8000, 90014 Oulu, Finland; 4 Department of Orthopaedics and Traumatology, Tampere University Hospital, Kuntokatu 2, 33520 Tampere, Finland

**Keywords:** Musculoskeletal system, Health care economics, Outcomes research

## Abstract

Due to the risk of adverse reactions to metal debris resulting from increased wear of the arthroplasty more than one million metal-on-metal (MoM) hip replacements worldwide are in active follow-up. Follow-up usually includes measurement of both whole blood cobalt (Co) and chromium (Cr) concentrations. Our experience is that Cr is seldom independently elevated. We wanted to ascertain whether blood Cr measurements could be omitted from follow-up protocols without lowering the quality of follow-up. We identified 8438 whole blood Co and Cr measurements performed without or prior to revision surgery. When the cut-off levels 5 µg/L and 7 µg/L were used, Cr was independently elevated in only 0.5% (95% confidence interval, CI, 0.3 to 0.6) and 0.2% (CI 0.1 to 0.3) of the measurements. The models with continuous variables showed that the higher the blood metal concentrations are the lower the percentage of measurements with Cr higher than Co. Our results suggest that whole blood Cr is very rarely independently elevated and therefore the authorities should consider omitting Cr measurements from their screening guidelines of MoM hip replacements. We believe this change in practice would simplify follow-up and lead to cost savings without decreasing the quality of follow-up.

## Introduction

After the acknowledgement of adverse reactions to metal debris and unacceptably high revision rates among metal-on-metal (MoM) hip replacements a decade ago, several national and international authorities published follow-up guidelines for MoM hip replacements that included clinical evaluation, imaging, and whole blood or serum metal ion measurements^1–4^. In 2012, it was estimated that there were nearly one million MoM hip replacements in the United States (US), and the number was still increasing^5^. In the United Kingdom (UK), the number of MoM hip replacements is around 70,000^6^, and in Australia it is around 35,000^3^. As an approximation, the figure of 1.5 million MoM hip replacements worldwide is often quoted^7^.

In their latest guidelines, both the UK Medicines and Healthcare products Regulatory Agency (MHRA)^8^ and the Australian Therapeutic Goods Administration (TGA)^3^ recommend the testing of both whole blood cobalt (Co) and chromium (Cr) for all patients with MoM hip replacements. Furthermore, the Scientific Committee on Emerging and Newly Identified Health Risks (SCENIHR) of the European Commission recommends blood metal ion measurements from all patients with a large head size MoM total hip arthroplasty (THA) implant or hip resurfacing^4^. Conversely, the US Food and Drugs Administration (FDA) recommends measuring Co from symptomatic patients, but does not consider the measurement of Cr a necessity^2^. Moreover, whereas most guidelines recommend annual systematic follow-up for the life of the implant for high risk implant/patient groups^3,4,8,9^, the FDA does not recommend the metal ion testing of asymptomatic patients with no abnormal clinical or radiographic signs^2^.

The cost of the measurements varies depending on the institution, but the prices mentioned in the literature range, for example, from $50 to $62 per measurement^10,11^. During the follow-up of patients at our institution, we have noticed that Cr is seldom higher than Co at concentrations above the suggested cut-off values of 5 µg/L^12^ or 7 µg/L^1^, and even when Cr is higher, Co is also found to be elevated. Atrey et al. reported that none of their 469 MoM patients had an independently elevated serum Cr level, and that they have stopped measuring Cr and now only measure Co which saves $18 per measurement^10^. Also Matharu et al. suggested that measuring Co only is sufficient and with their laboratory prices the saving per measurement is $15.40^13^.

We conducted a study to analyze whether Cr measurements are needed, or whether standard follow-up protocols could be run by only including blood Co measurements. The aims of the study were to answer the following questions: 1) What percentage of whole blood Co and Cr measurements have higher Cr than Co and 2) In how many measurements is Cr independently elevated when the cut-off values of 5 µg/L or 7 µg/L are used?

## Materials and methods

The study was approved by the Tampere University Hospital Ethics Committee (approval ID R11006 and R11196). Based on Data Protection Act in Finland, informed consent was not needed in this study because only retrospective analysis of the data collected during standard care was performed and this study did not include any additional contacts or tests on the patients. The need for informed consent was waived by the Tampere University Hospital Ethics Committee. The ethical guidelines of the World Medical Association’s Declaration of Helsinki were followed.

After the recall of the Articular Surface Replacement (ASR, Depuy Orthopaedics, Warsaw, IN)^14^ and reports of its poor survival in the Australian Orthopaedic Association Joint Replacement Registry^15^, our institution launched an intensified screening program for all ASR MoM hip replacements in 2010. Screening included whole blood Co and Cr measurements, physical examination, and cross-sectional imaging with magnetic resonance imaging as the primary imaging modality. Later, in 2012, other MoM hip replacement brands were also included in the screening program.

Blood samples were drawn from the antecubital vein with a vacutainer system. The first 10 mL was used for other analyses to prevent Co and Cr contamination from the needle. Whole blood Co and Cr measurements were performed with dynamic reaction cell inductively coupled plasma mass spectrometry (Agilent 7500cx or 8800, Agilent Technologies, Santa Clara, CA, USA).

We included all patients with a MoM hip replacement implanted at our institution. For these patients, all Co and Cr measurements available in our laboratory database were retrieved. The patients with MoM hip replacements were identified from our hospital database. The data scientist then deidentified the data before continuing to further data retrieval and analyses. No referral patients were included.

### Statistics

The percentage of measurements with Cr higher than Co was calculated. As there are differences in metal ion release from stemmed THAs and hip resurfacings^16^, subgroup analyses were performed for the measurements from patients with stemmed THA (unilateral or bilateral) and for those measurements from patients with hip resurfacing (unilateral or bilateral). Measurements from patients with THA in one hip and resurfacing in the other hip were not analyzed as a separate group. As it has been previously reported that Cr might remain elevated after revision surgery^17^, measurements performed after revision surgery were excluded from most of our analysis. The analyses without exclusion are shown in Supplement 1. In patients with bilateral MoM hip replacement, the exclusion was done after the first revision, and therefore even if they had one MoM hip replacement remaining, the measurements taken after the revision were omitted. To analyze the percentage of measurements with independently elevated Cr, we reported the number of measurements with Cr higher than Co in those cases where either Co or Cr exceeded the cut-off value. In the analysis, we used two commonly used cut-off values, 5 µg/L by Hart et al.^12^ and 7 µg/L by the UK MHRA^1^. The Wilson method was used for calculating 95% confidence intervals (CI).

Because several other cut-off values have been suggested^13,18–20^ and there is no consensus on the best cut-off value or even whether cut-offs values should be used at all, we also analyzed the concentrations as continuous variables. The analysis was performed by ranking the measurements by whole blood Co concentration and plotting the percentage of measurement with Cr higher than Co among the measurements, where Co is above a specified value. Further, Bland–Altman plots were drawn to describe the differences between Co and Cr levels. Plots were drawn separately for all measurements, for measurements from patients with stemmed THA, and for measurements from patients with hip resurfacing. Because our study included repeated measurements from some of the patients, we performed the main analyses with inclusion of only one measurement per patient (most recent measurement before/without revision surgery) as sensitivity analysis and presented them in Supplement 2.

The analyses were performed with R (Version 3.5.1)^21^.

## Results

At our institution, 2520 patients (3013 hips) were implanted with a MoM hip replacement between November 1999 and February 2012. We identified a total of 10,962 Co–Cr measurements drawn from 2254 patients. Patient demographics are presented in Table 1. Whole blood Co and Cr measurements were performed between January 2008 and February 2020. The mean time from primary surgery to the whole blood metal ion measurement was 8.3 years (SD 3.4). Of these measurements, 6667 were from patients with unilateral or bilateral THA, 4101 from patients with unilateral or bilateral hip resurfacing, and 194 were from patients with THA in one hip and hip resurfacings in the other. Cr was higher than Co in 4958 (45%, CI 44–46) of the 10,962 measurements. Among THAs, Cr was higher in 2170 (33%, CI 31–34) and among hip resurfacings in 2714 (66%, CI 65–68) (Table 2).

**Table 1:** Demographics of the patients from whom the whole blood cobalt and chromium samples were drawn.

	All patients with MoM hips	MoM total hip arthroplasty	MoM hip resurfacing
No. of patients	2254	1335	890
No. of paired measurements	10,962	6667	4101
Median Co (range, μg/L)	1.7 (0.1–225)	2.5 (0.1–192)	1.2 (0.1–225)
Median Cr (range, μg/L)	1.6 (0.1–125)	1.7 (0.1–115)	1.4 (0.1–125)
Age at surgery, median (range)	68 (16–93)	70 (19–93)	63 (16–91)
Gender (female/male, n, %)	1037 (46%) / 1217 (54%)	667 (50%) / 668 (50%)	347 (39%) / 543 (61%)

MoM, metal-on-metal; Co, cobalt; Cr, chromium.

**Table 2:** Percentages of paired measurements with higher whole blood chromium value compared to cobalt.

Type of implant	Total number of measurements			Measurements before or without revision			Before or without revision and Co or Cr ≥ 5 µg/L				Before or without revision and Co or Cr ≥ 7 µg/L				
	n	Cr higher n/% (CI)	Cr lower or equal n/% (CI)	N*	Cr higher n/% (CI)	Cr lower or equal n/% (CI)	n	Cr higher n/% (CI)	Cr lower or equal n/% (CI)	Cr ≥ 5 and Co < 5 n/%/ % of N* (CI)	n		Cr higher n/% (CI)	Cr lower or equal n/% (CI)	Cr ≥ 7 and Co < 7 n/%/ of N* (CI)
All	10,962	4958/ 45% (44–46)	6004/ 55% (54–56)	**8438**	3543/ 42% (41–43)	4895/ 58% (57–59)	2024	79/ 3.9% (3.1–4.8)	1945/ 96% (95–97)	38 /1.9% (1.4–2.6) /**0.5% (0.3–0.6)**	1362		27/ 2.0% (1.4–2.9)	1335/ 98% (97–99)	13 /1.0% (0.6–1.6) /**0.2% (0.1–0.3)**
THA	6667	2170/ 33% (31–34)	4497/ 67% (66–69)	**4769**	1194/ 25% (24–27)	3575/ 75% (74–76)	1712	18/ 1.1% (0.7–1.7)	1694/ 98.9% (98.3–99.3)	9 /0.5% (0.3–1.0) /**0.2% (0.1–0.4)**	1178		6/ 0.5% (0.2–1.1)	1172/ 99.5% (98.9–99.8)	4 /0.3% (0.1–0.9) /**0.1% (0.03–0.2)**
Hip resurfacing	4101	2714/ 66% (65–68)	1387/ 34% (32–35)	**3543**	2322/ 66% (64–67)	1221/ 34% (33–36)	259	58/ 22% (17–28)	201/ 78% (72–82)	27 /10% (7.2–15) /**0.8% (0.5–1.1)**	145		19/ 13% (8.6–20)	126/ 87% (80–91)	8 /5.5% (2.8–11) /**0.2% (0.1–0.5)**

Co, cobalt; Cr, chromium; CI, 95% confidence interval; THA, total hip arthroplasty.

In columns “measurements before or without revision” the Co and Cr measurements performed after a revision surgery are excluded because it is known that whole blood Cr may remain elevated longer than Co after revision surgery^17^.

A total of 8438 measurements were performed either prior to revision surgery or on patients who had not undergone revision (2524 measurements after revision). Among these, Cr was higher in 3543 (42%, CI 41 to 43) of the measurements. Cr was higher in 1194 (25%, CI 24 to 27) of the measurements performed on patients with stemmed THA and in 2322 (66%, CI 64 to 67) of those with hip resurfacing.

Among the measurements drawn from those who had not undergone revision surgery, either Co or Cr was above the 5 µg/L cut-off^12^ in 2024 (24%, CI 23 to 25) measurements and above the 7 µg/L^1^ cut-off in 1362 (16%, CI 15 to 17) measurements. Cr was higher than Co in 79 (3.9%, CI 3.1 to 4.8) of the measurements, where Co or Cr was above 5 µg/L and in 27 (2.0%, CI 1.4 to 2.9), where Co or Cr was above 7 µg/L. Independently elevated Cr values (Cr above the cut-off and Co below the cut-off) were seen in 38 (0.5%, CI 0.3 to 0.6, 5 µg/L cut-off) or 13 (0.2%, CI 0.1 to 0.3, 7 µg/L cut-off) of the 8438 measurements (Table 2). The same analyses without the exclusion of the measurements after revision surgery resulted in higher percentage of Cr being independently elevated (Supplement 1).

**Figure 1:**
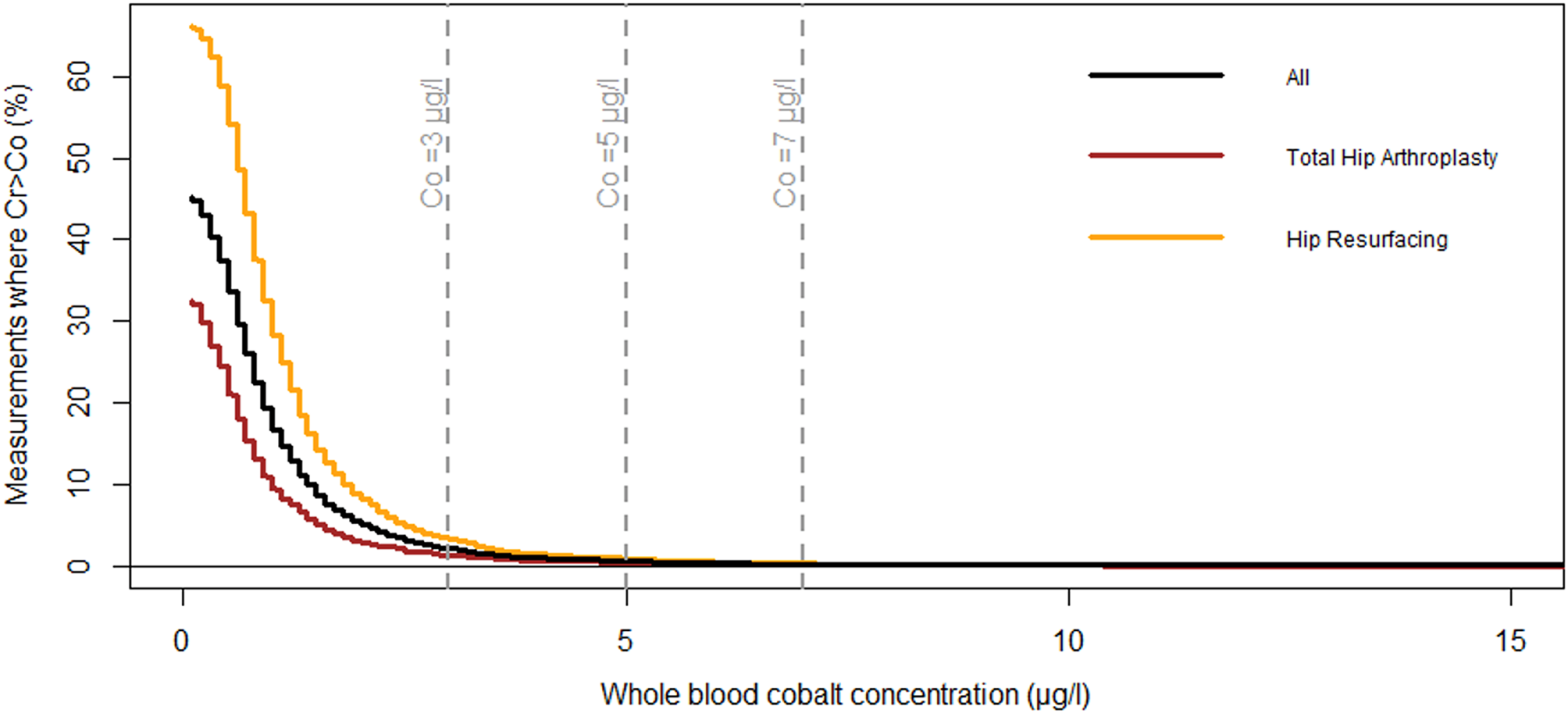
The relationship between whole blood cobalt (Co) concentration and the percentage of measurements where chromium (Cr) concentration is higher than Co. At a certain whole blood Co value, the curve tells us what percentage of the measurements with Co above the value have higher whole blood Cr than Co. “All” corresponds to all measurements performed without or before revision surgery. Figure drawn with R (Version 3.5.1)^21^.

**Figure 2:**
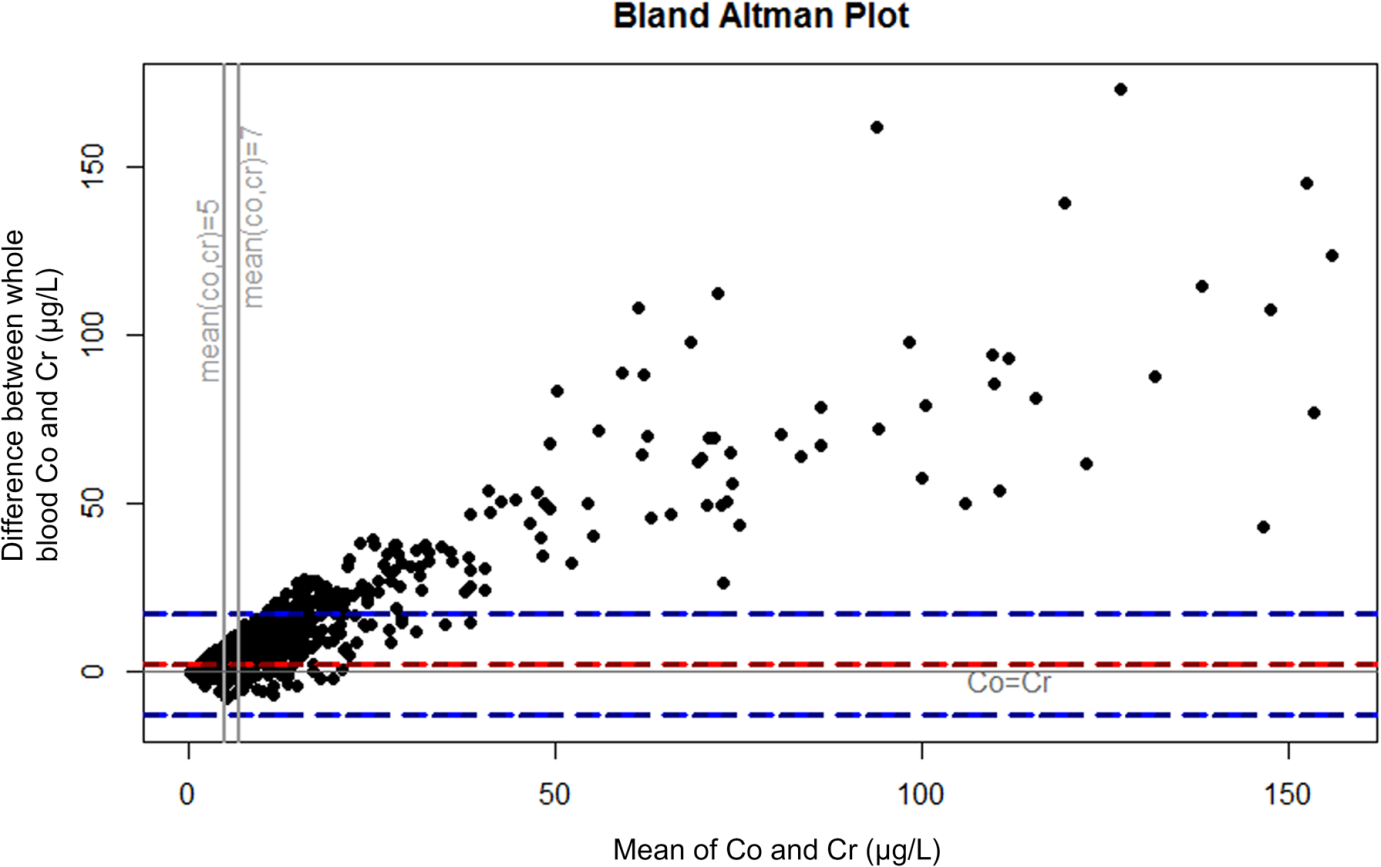
Bland–Altman plot representing the difference between whole blood Co and Cr in relation to the mean of whole blood Co and Cr (µg/L). In the measurements below zero the whole blood Cr is higher than Co. The red line represents the mean difference and blue lines 95% confidence intervals. Measurements before/without revision surgery from all patients with MoM hip replacement. Figure drawn with R (Version 3.5.1)^21^.

**Figure 3:**
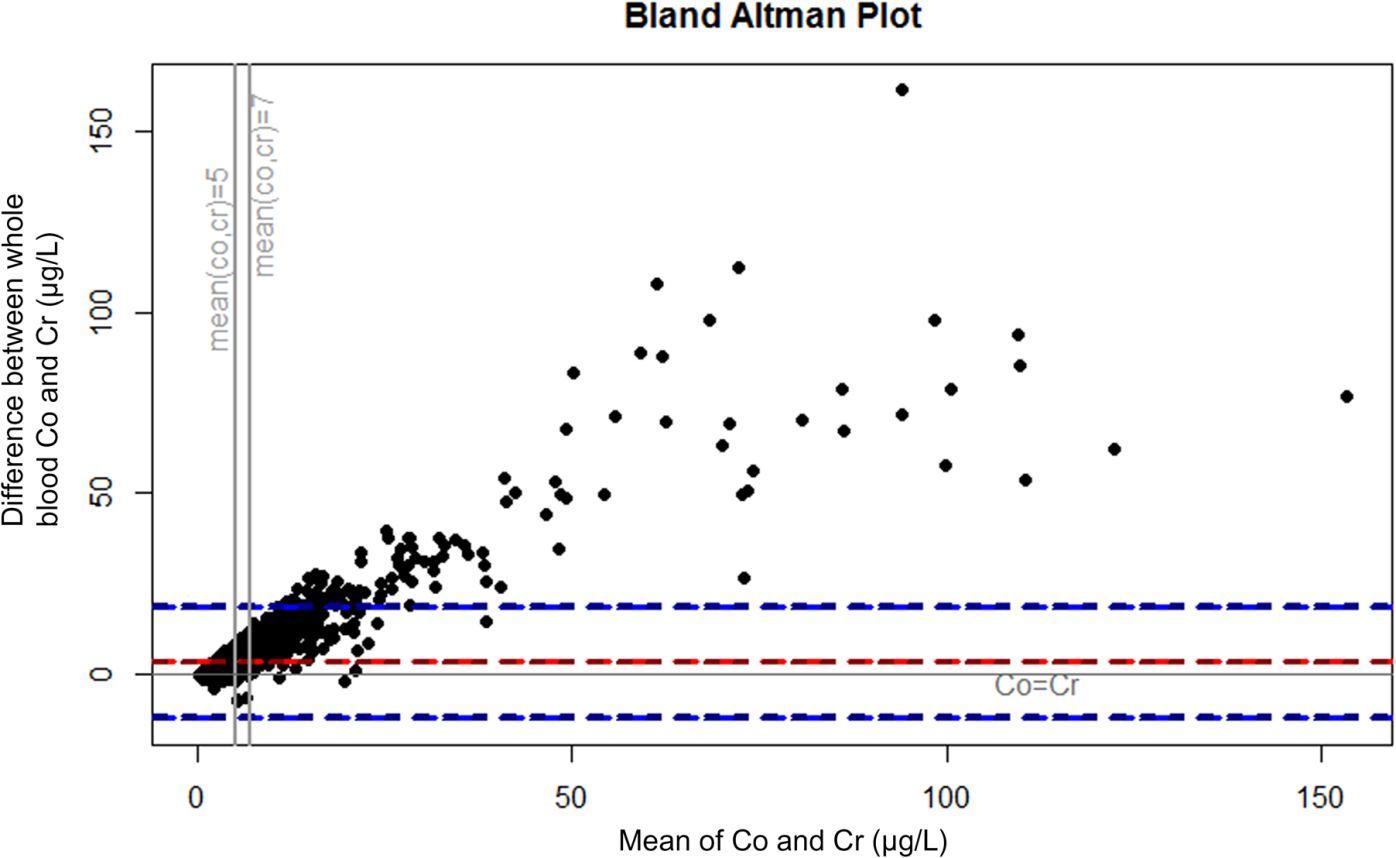
Bland–Altman plot representing the difference between whole blood Co and Cr in relation to the mean of whole blood Co and Cr (µg/L) in patients with unilateral or bilateral stemmed total hip arthroplasty. In the measurements below zero the whole blood Cr is higher than Co. The red line represents mean difference and blue lines 95% confidence intervals. Measurements before/without revision surgery. Figure drawn with R (Version 3.5.1)^21^.

**Figure 4:**
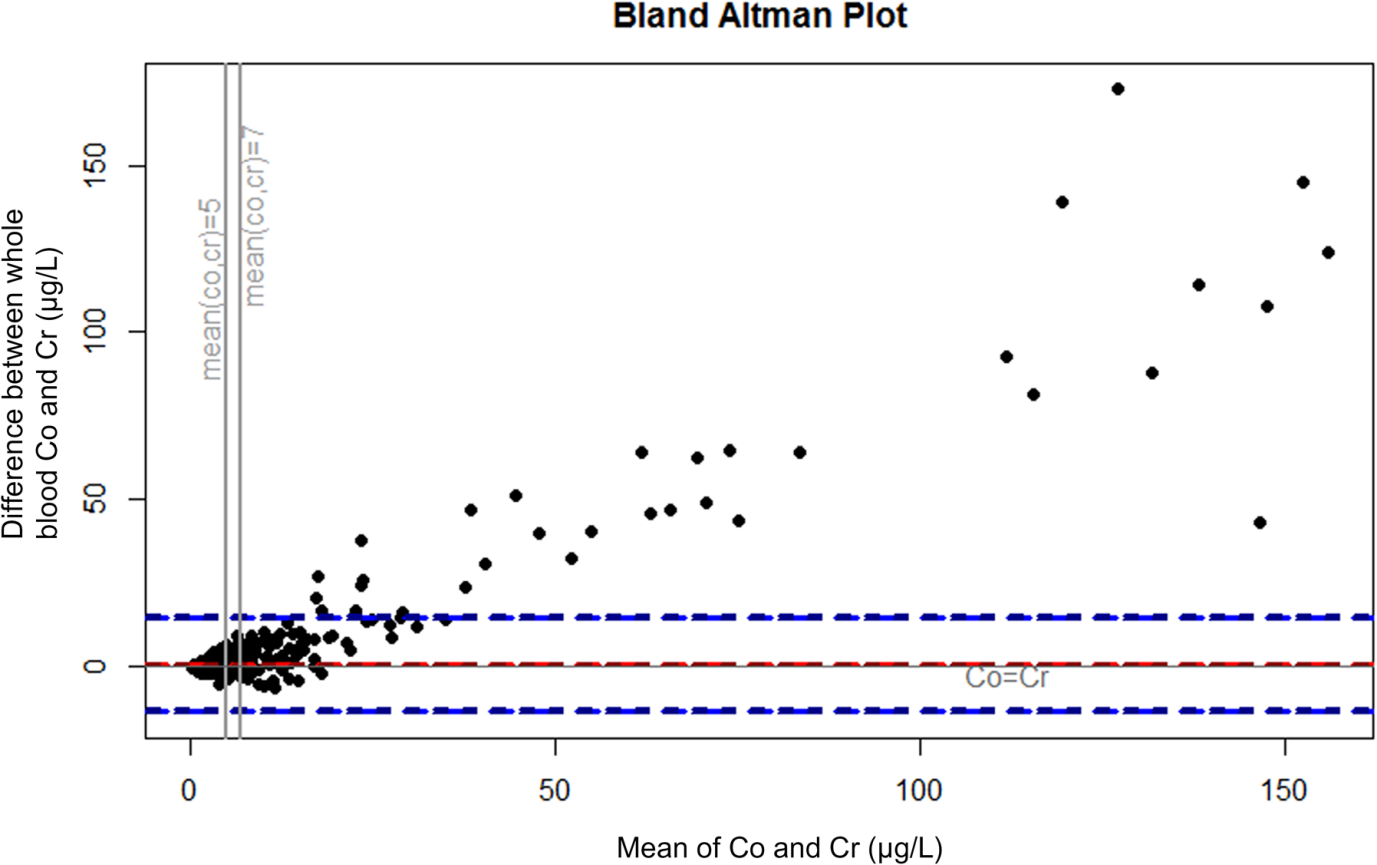
Bland–Altman plot representing the difference between whole blood Co and Cr in relation to the mean of whole blood Co and Cr (µg/L) in patients with unilateral or bilateral hip resurfacings. In the measurements below zero the whole blood Cr is higher than Co. The red line represents mean difference and blue lines 95% confidence intervals. Measurements before/without revision surgery. Figure drawn with RStudio R (Version 3.5.1)^21^.

The very small percentage of the measurements with Cr higher than Co at higher whole blood Co concentrations is presented in Fig. 1. Bland–Altman plots for all measurements (Fig. 2), measurements from THAs (Fig. 3), and from hip resurfacings (Fig. 4) also demonstrate that measurements with Cr higher than Co are only seen at relatively low values. At very high concentrations, there are virtually no measurements where Cr is higher than Co. Sensitivity analyses performed with inclusion of only one measurement per patient resulted in an even smaller percentage of independently elevated whole blood Cr (Supplement 2 and 3).

## Discussion

The results of our study show that at whole blood Co and Cr concentrations above the cut-offs of 5 µg/L and 7 µg/L, Cr measurements add very little to Co measurements in detecting high wearing MoM hip replacements. At lower levels, there are more measurements where Cr is higher than Co, but we do not consider these to be relevant. The median whole blood Cr 1.5 µg/L and Co 0.5 µg/L have been reported as background concentrations analyzed from 3042 blood samples donated to a local transfusion center^20^. We believe that in asymptomatic patients with no evidence of osteolysis, loosening, or fractures in x-ray, only slightly (independently) elevated Cr would not lead to revision at virtually any institution.

To the best of our knowledge, this is the largest number of Co-Cr measurements published to date from a single institution. The strength of our study is the unselected patient group. Our hospital is a large primary center, with a high volume of approximately 2000 primary hip arthroplasties per year, performing more than 95% of the hip arthroplasties in our region. Our public health care system ensures that this patient group represents the overall MoM population in terms of gender, age, socioeconomic status, and lifestyle. From the implant brand point of view, we consider our results to be very generalizable. When compared with the two largest hip arthroplasty registers (Supplement 4) Australia^22^ and the UK^6^, the distribution of MoM THA brands at our institution is similar to that of the Australian registry. Moreover, the two major THA brands in the UK registry are the two most used stemmed MoM THA implant brands at our institution. Although Adept and Cormet resurfacings were not used at our institution, both of which were commonly used in Australia (10% and 4%) and in UK (10% and 10%), the other commonly used resurfacing brands are well represented in our study group.

We did exclude measurements performed after revision from our main analyses as our previous study showed that Cr might remain elevated after revision surgery. We think that this exclusion is justified, as when Table 2 and Supplement 1 are compared, it can be seen that measurements after revision are more often related with independently elevated Cr. Although there are relatively many revisions among patients with MoM hip replacements, majority of patients still have well-performing implants. Thus, we think that one should aim to simplicity and cost reductions especially in follow-up of these patients, who still comprise the largest MoM patient group and will be going through multiple repeated measurements. Our current study does not take a stance on the post-MoM-revision follow-up. The European consensus statement advises against routine metal ion measurements after revision as there are no effective interventions available even if the ions remain elevated after revision^9^. Even if some institutions wish to monitor Co and Cr after revision until they are at normal level, this comprises a relatively small patient group and the follow-up can be discontinued when Co/Cr are at normal level, as the source of ions has been removed. Therefore, the need for cost savings is clearly smaller than in unrevised patients who may have to go through repeated Co and Cr measurements for decades. Longitudinal changes of Co and Cr were not included in this article, as it is such a vast aspect with much discussion needed about survivorship bias due to high prevalence of revisions. A separate study will be published about longitudinal changes.

Whole blood metal ion measurements are included in all major follow-up guidelines^2–4,8,9^. The UK MHRA and the TGA mention both Co and Cr^3,8^, whereas the FDA does not consider Cr measurements a necessity^2^. To date, however, no strong evidence has been presented that shows that one of these practices would be better than the other. Moreover, some institutions have omitted Cr due to their experience of negligible benefit^10^. Although MoM hip replacements are not the huge controversy they were a decade ago, it should be noted that the majority of MoM hip replacements still remain in situ*.* Based on the revision rates reported by UK National Joint Replacement Registry (15-year cumulative percentage of 24% for stemmed MoM THAs and 15% for resurfacings)^6^ and the Australian Orthopaedic Association Registry (15-year cumulative percentage revision of 29% for stemmed MoM THAs with head size > 32 mm and 12% for resurfacings)^22^, it is likely that over one million out of the estimated 1.5 million MoM hips implanted worldwide^7^ still remain in situ and are under follow-up as recommended by the majority of the guidelines^2–4,9^. Annual follow-up for the life of the implant is recommended for high risk implant/patient groups by most guidelines^3,4,8,9^. However, the FDA does not recommend the metal ion testing of asymptomatic patients with no abnormal clinical or radiographic signs^2^. Low risk MoM implants can be followed at less frequent intervals^8^ or even at intervals similar to those of conventional hip replacements^3,4,9^. The follow-up of MoM hip replacements has been criticized for being neither evidence based nor cost effective^7,11^. Based on the savings of $15–18 per measurement when Cr is omitted reported by Atrey et al. and Matharu et al.^10,13^,we believe that vast sums of money could be saved worldwide during the next decades by omitting Cr measurements from the routine follow-up of MoM patients, without decrease in the quality of the follow-up.

There is a large variety of cut-off values proposed for Co and Cr measurements. UK MHRA has been using blood metal ion level of 7 µg/L as a cut-off for indicating need for closer follow-up and cross-sectional imaging^8^. Their reports do not state references on which the cut-off is based on. Another commonly used cut-off of 5 µg/L is based on a study of 88 patients with MoM hip patients waiting for revision due to unexplained reason compared to 88 patients with well-functioning MoM hips^12^. That study found that 7 µg/L limit has insufficient sensitivity, and suggested 5 µg/L as blood Co/Cr limit to detect failure of MoM hip. A study comparing plasma Co and Cr concentrations to MRI findings in symptomatic patients concluded that sensitivity of 7 µg/L limit offers insufficient sensitivity and Co/Cr measurements should not be used as sole screening test^23^. Whole blood cut-off of Co 4.5 µg/L has been proposed to predict increased wear^20^, and serum levels Co 4.0 µg/L and 4.6 µg/L for unilateral hip resurfacings to predict poor-/well-functioning of the implant, and 5.0 µg/L and 7.4 µg/L for bilaterals, respectively^18^. A consensus statement from US suggested 3 µg/L as an upper limit for low risk and 10 µg/L as lower limit for high risk (without specification of whole blood/plasma/serum), both of which seem to be arbitrary limits^19^. More recently, lower, implant specific whole blood Co cut-offs have been presented to differentiate between those with ARMD in revision or imaging and those with not^13,24^. There are also other suggested cut-offs not stated here. Almost every study published about Co and Cr cut-offs stresses that metal ion measurements should not be used as sole indicator of ARMD, and we do agree with them. Therefore, we included an analysis of metal ion concentrations as a continuous variable as we believe that there is no such thing as a universally optimal cut-off. Still, we wanted to include analyses with two widely used cut-offs of 5 µg/L and 7 µg/L to better demonstrate to those readers who use these cut-offs that how seldom Cr measurements provide any additional value.

Both whole blood and plasma/serum measurements are used for follow-up of MoM hip replacements, although guidelines recommend using whole blood measurements^2,4,8^. There are few studies about interchangeability of whole blood and plasma/serum measurements of Co and Cr, all of which came into a conclusion than whole blood and plasma/serum measurements cannot be used interchangeably^20,25–27^. Two of these studies^25,27^ stated that the values are not interconvertible, meaning that plasma/serum values cannot be reliably converted to whole blood value with a conversion factor, while one^26^ suggested that conversion factor could be used. These studies observed higher discrepancy between plasma/serum and blood ion levels at higher concentrations^20,25,26,28^. The difference between plasma/serum and blood values is larger in Cr compared to Co, with higher Cr concentration in plasma/serum^25–28^. As the conclusions in this study are based on whole blood Cr concentrations being rarely independently elevated, because of tendency for relatively higher plasma/serum Cr concentration the results might be different if we had used plasma/serum measurements. We do not know how large percentage of the measurements are performed from plasma/serum worldwide.

Co/Cr ratio has been proposed as an additional way to measure taper corrosion in MoM THAs^16^, and Co/Cr ratio is reported to be higher in THAs compared to resurfacings^29,30^. Few studies have explored the clinical use of Co/Cr ratio for detecting MRI or revision confirmed ARMD by examining receiving operating characteristic (ROC) curves, and none of them found Co/Cr ratio to be superior compared to measuring Co alone^13,29,31^. We do think that it is not even relevant whether or not high Co/Cr ratio predicts the presence of ARMD. Increasing Co/Cr ratio describes a disproportional increase of Co compared to Cr, which is very often the case as seen in our Figs. 2,3and4. If an institution would change their practice to measuring Co only, they would still detect the patients in which the Co is increasing, despite not calculating Co/Cr. Increasing Co/Cr ratio raises concerns due to increasing Co, not because of decreasing Cr. We are currently moving from mid-term to long-term follow-up and majority of the remaining MoM hips are still performing well. If an institution wishes to use Co/Cr ratio for pre-revision planning to estimate whether the bearing surface or the trunnion is more likely to be a problem, additional Co and Cr measurement can still be used for those few patients who are under consideration for revision surgery or with suspicion of trunnion problematics, while simplifying the follow-up of the vast majority. And it must also be noted that pre-revision Co/Cr ratio has not been shown to reliably predict the severity of taper corrosion. Thus, it does not help the surgeon to decide beforehand, whether the stem also needs to be exchanged if it is well-fixed in pre-revision radiographs. In practice, that decision is still made perioperatively no matter how high the blood metal ion values or the Co/Cr ratio are.

## Conclusion

Our study provides robust evidence that whole blood Cr is very rarely elevated without whole blood Co also being elevated. As current guidelines either place equal value on Co and Cr in decision making, or even stress the importance of blood Co concentration, the authorities should consider omitting Cr measurements from their screening guidelines of MoM hip replacements. Recent studies have reported that measuring only Co would result in savings of $15–18 per measurement. Considering the large number of patients with MoM hip replacements still in follow-up going through repeated measurements, this simple change in clinical practice could result in large cost savings.

## Supplementary Information

Supplementary Information.

## Data Availability

The datasets generated and analyzed during the current study can’t be made publicly available due to containing identifiable information of our patients (the policy by our hospital district). We will be happy to provide additional analyses requested by the readers of the article, for example to be used in systematic review articles. On a reasonable request we can provide the whole blood Co and Cr measurement data without other clinical information (which could be combined to identify the patients).
