# Supplementary material for: Whole blood chromium concentration is very rarely elevated independently of whole blood cobalt
Source: Sci Rep. 2021 Jun 11;11:12352. doi: 10.1038/s41598-021-91512-9 (PMC8196186; doi:10.1038/s41598-021-91512-9)
Supplement: Supplementary file 1 — Supplementary Information. [file 41598_2021_91512_MOESM1_ESM.pdf]

Supplementary Files

Whole blood chromium concentration is very rarely elevated independently of whole blood cobalt

This work was performed at Coxa Hospital for Joint Replacement and Faculty of Medicine and Health Technologies, Tampere University

Supplement 1. Percentages of measurements with higher whole blood chromium value compared to cobalt without exclusion of the measurements performed after revision surgery.

Type of implant	Total number of measurements			Co or Cr ≥ 5 µg/L				Co or Cr ≥ 7 µg/L			
	N*	Cr higher n/% (CI)	Cr lower or equal n/% (CI)	n	Cr higher n/% (CI)	Cr lower or equal n/% (CI)	Cr≥5 and Co<5 n/%/ **% of N*** (CI)	n	Cr higher n/% (CI)	Cr lower or equal n/% (CI)	Cr≥7 and Co <7 n/%/ **of N*** (CI)
All	**10962**	4958/ 45% (44-46)	6004/ 55% (54-56)	2651	225/ 8.4% (7.5-9.6%)	2426/ 92% (90-93)	156 /5.9% (5.1-6.8) **/1.4% (1.2-1.7)**	1788	122/ 6.8% (5.7-8.1)	1666/ 93 % (92-94)	99 /5.5% (4.5-6.7) /**0.9% (0.7- 1.1)**
THA	**6667**	2170/ 33% (31-34)	4497/ 67% (66-69)	2197	102/ 4.6 % (3.8-5.6)	2095/ 95% (94-96%)	73 /3.3% (2.7-4.2) **/1.1% (0.9-1.4)**	1508	65/ 4.3% (3.4-5.5)	1443/ 96 % (95-97)	57 /3.8% (2.9-4.9) /**0.9% (0.7-1.1)**
Hip resurfacing	**4101**	2714/ 66% (65-68)	1387/ 34% (32-35)	378	112/ 30% (25-34)	266/ 70% (66-75)	74 /20% (16-24) **/1.8% (1.4-2.3)**	226	52/ 23% (18-29)	174/ 77% (71-82)	39 /17% (13-23) /**1.0% (0.7-1.3)**

Co, cobalt; Cr, chromium; CI, 95% confidence interval; THA, total hip arthroplasty.

Supplement 2. Percentages of measurements with higher whole blood chromium value compared to cobalt with the exclusion of the measurements performed after revision surgery. Only one (the most recent) measurement per patient.

Type of implant	Total number of measurements			Co or Cr ≥ 5 μg/L				Co or Cr ≥ 7 μg/L			
	N*	Cr higher n/% (CI)	Cr lower or equal n/% (CI)	n	Cr higher n/% (CI)	Cr lower or equal n/% (CI)	Cr≥5 and Co<5 n/%/ **% of N*** (CI)	n	Cr higher n/% (CI)	Cr lower or equal n/% (CI)	Cr≥7 and Co <7 n/%/ **of N*** (CI)
All	**2153**	919/<br>43%<br>(41-45)	1234/<br>57%<br>(55-59)	645	19/<br>2.9%<br>(1.9-4.6)	626/<br>97%<br>(95-98)	5<br>/0.8% (0.3-1.8)<br>**/0.2% (0.01-0.5)**	496	11/<br>2.2%<br>(1.2-3.9)	485/<br>98 %<br>(96-99)	4<br>/0.8% (0.3-2.1)<br>/**0.2% (0.07- 0.5)**
THA	**1267**	328/<br>26%<br>(24-28)	936/<br>74%<br>(71-76)	546	7/<br>1.3%<br>(0.6-2.6)	539/<br>99%<br>(97-99%)	2<br>/0.4% (0.1-1.3)<br>/**0.2% (0.04-0.6)**	419	2/<br>0.5%<br>(0.1-1.7)	417/<br>99.5%<br>(98-99.9)	0<br>/0.0%<br>/**0.0%**
Hip resurfacing	**860**	587/<br>68%<br>(65-71)	273/<br>32%<br>(29-35)	85	11/<br>13%<br>(7.4-22)	74/<br>87%<br>(78-93)	3<br>/3.5% (1.2-9.9)<br>/**0.4% (0.1-1.0)**	65	8/<br>12%<br>(6.4-22)	57/<br>88%<br>(78-94)	4<br>/6.2% (2.4-15)<br>/**0.5% (0.2-1.2)**

Co, cobalt; Cr, chromium; CI, 95% confidence interval; THA, total hip arthroplasty.

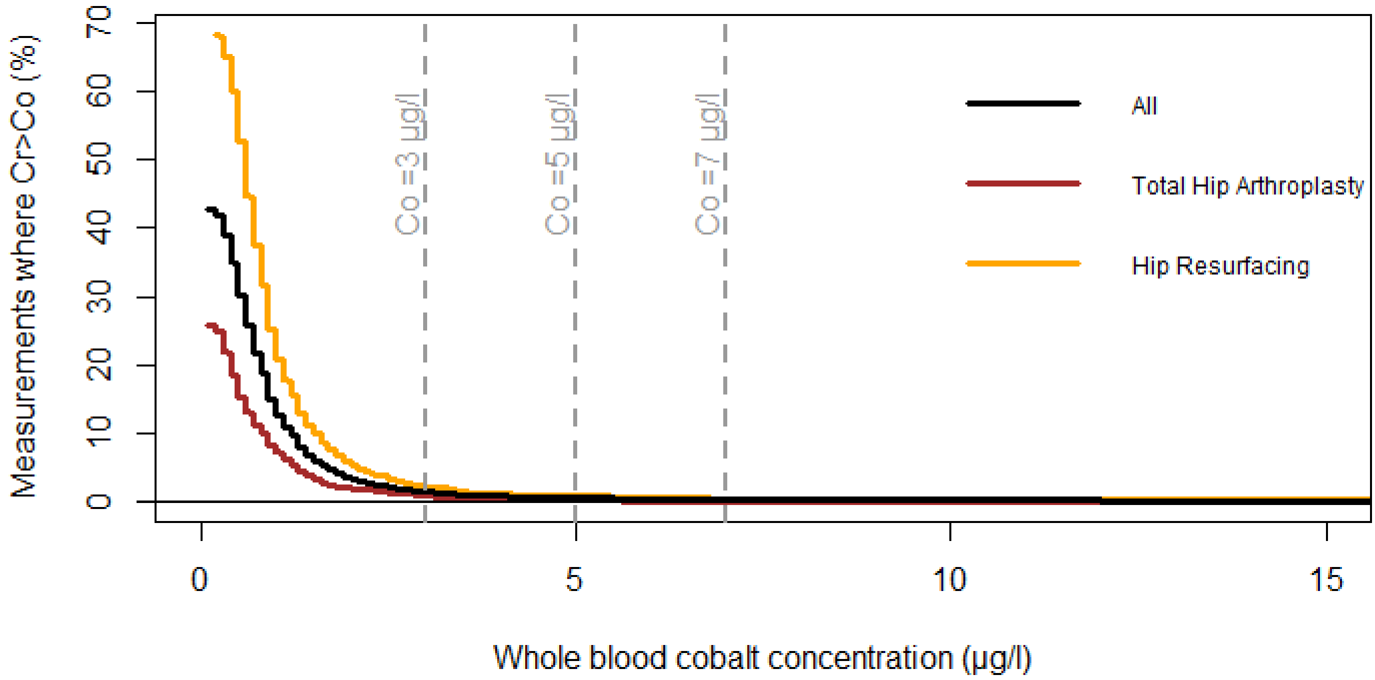

Supplement 3. Sensitivity analysis with the inclusion of only one measurement per patient (the most recent measurement before/without revision, a total of 2153 measurements). The relationship between whole blood cobalt (Co) concentration and the percentage of measurements where chromium (Cr) concentration is higher than Co. At a certain whole blood Co value, the curve tells us what percentage of the measurements with Co above that value have higher whole blood Cr than Co. "All" corresponds to all measurements performed without or before revision surgery. Figure drawn with RStudio (Version 1.1.456) [21].

Supplement 4. Percentage of different MoM implant brands used at the Australian Orthopaedic Association National Joint Replacement Registry (AOANJRR) (22), our institution, and in the National Joint Registry for England, Wales, Northern Ireland, the Isle of Man, and the States of Guernsey (UK NJR) (6).

AOANJRR		Our institution*		UK NJR	
THA	%	THA	%	THA	%
ASR	41	ASR	34	ASR	19
Pinnacle	18	Pinnacle	28	Pinnacle	81
BHR	12	BHR	8		
ReCap	9	ReCap	11		
M2A	5	M2A	5		
Durom	6	Durom	3		
Mitch	4	Mitch	1		
Bionik	4	R3	7		
		Other	3		

AOANJRR		Our institution*		NJR	
Resurfacing	%	Resurfacing		Resurfacing	
ASR	7	ASR	39	ASR	8
Adept	10			Adept	10
BHR	66	BHR	37	BHR	60
Bionik	1	Conserve	1	Conserve	4
Cormet	4			Cormet	10
Durom	5	Durom	15	Durom	5
Icon	1				
Mitch	6	Mitch	0.3		
ReCap	1	ReCap	6	ReCap	5
Other	1	Other	2		

THA, total hip replacement. The implant brands used in AOANJRR and/or UK NJR but not used at our institution are highlighted in blue. *Figures for our institution were calculated from all 3013 MoM hip replacements implanted at our institution.

ASR, Articular Surface Replacement (Depuy Warsaw, IN); Adept (*MatOrtho* Limited, Surrey, United Kingdom); BHR, Birmingham Hip Resurfacing, (Smith and Nephew, Memphis, TN); Bionik (*Eska* Implants, Lübeck, Germany); Conserve (Wright Medical, Memphis, TN); Cormet (Stryker, Mahwah, NJ); Durom (Zimmer, Warsaw, IN); M2A (Biomet, Warsaw, IN); Mitch (Stryker), Pinnacle (Depuy), R3 (Smith and Nephew) ReCap (Biomet).
